# Secondary syphilis sparing palms and soles, with pulmonary involvement

**DOI:** 10.1590/S1678-9946202567077

**Published:** 2025-11-03

**Authors:** María Pineda-Muñoz, Ayleen Rivera-Tenorio, Cindy Alejandra Bonilla-Sánchez, Mariana Botero, Javier Hernández-Moreno, Miguel C. Duarte-Villalba, Álvaro A. Faccini-Martínez

**Affiliations:** 1 Hospital Militar Central, Servicio de Dermatología, Bogotá D.C, Colombia; 2 Pontificia Universidad Javeriana, Programa de Especialización en Infectología, Bogotá D.C, Colombia; 3 Hospital Militar Central, Servicio de Patología, Bogotá D.C, Colombia; 4 Hospital Militar Central, Servicio de Infectología, Bogotá D.C, Colombia; 5 Universidad Militar Nueva Granada, Facultad de Medicina, Bogotá D.C, Colombia

**Keywords:** Secondary syphilis, Treponema pallidum, Lung diseases, Immunohistochemistry, Prozone phenomenon

## Abstract

Secondary syphilis can compromise many organs and is widely known for manifesting with classic signs, such as a rash that involves palms and soles along with mucosal lesions. The absence of these manifestations, along with false negative serological tests, as seen with the prozone phenomenon, poses a difficult challenge for the clinician. When skin lesions are visible, a biopsy for pathological description and immunohistochemistry for *Treponema pallidum* may help aid in the confirmation and certainty of the diagnosis. Here, we report a case of secondary syphilis with atypical manifestations.

## INTRODUCTION

Secondary syphilis can compromise many organs and is widely known for manifesting with classic signs such as a rash that involves palms and soles along with mucosal lesions. Up to 15%–30% of cases may present atypical cutaneous findings^1^, and in 0.5%–2% of secondary syphilis cases the prozone phenomenon is described yielding false-negative non-treponemal tests^2-4^, delaying diagnosis and timely treatment for the patient. Therefore, other diagnostic tools such as immunohistochemistry for *Treponema pallidum* of skin specimens are valuable for diagnosis confirmation with specificity approaching 100%^5,6^. Here, we report a case of secondary syphilis with atypical manifestations.

### Ethics

Ethics committee process Nº 272025. Written informed consent was obtained from the patient.

## CASE REPORT

**Figure 1:**
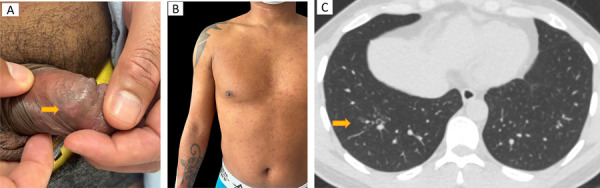
(A) discrete annular scar on the penis shaft; (B) Numerous erythematous macules, papules, and nodules were distributed throughout the neck, anterior thorax, abdomen, back, and superior extremities, sparing palms and soles; (C) Tree-in-bud pattern in the posterior basal segment of the right lower lobe.

On April 22, 2025, a 32-year-old male marine who had recently relocated to Bogota city, capital of Colombia, showed up to the emergency department at Hospital Militar Central. He reported a two-week history of bilateral testicular pain, followed by three days of fever and the appearance of a maculopapular rash on the anterior thorax and upper extremities. He also described a one-month history of constitutional symptoms, including a 10 kg weight loss, asthenia, and adynamia. He had bilateral inguinal adenopathy on physical examination, a discrete annular scar on the penile shaft, and widespread erythematous macules, papules, and nodules on the neck, anterior thorax, abdomen, back, and upper extremities (Figures 1A and 1B). No lesions were seen compromising mucous or palms and soles. Basic laboratory investigations were unremarkable, including complete blood cell count with differential, basic metabolic panel, and liver function tests. A fourth-generation HIV test was negative.

Throughout the hospital stay, the patient manifested chronic cough and night sweats, therefore, a chest computed tomography (CT) scan was performed identifying a Tree-in-bud pattern in the posterior basal segment of the right lower lobe (Figure 1C).

**Figure 2:**
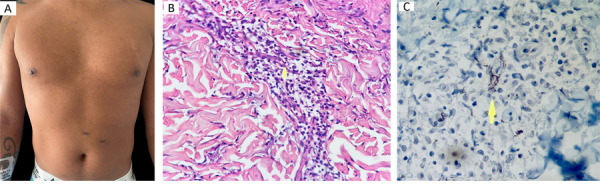
(A) Attenuation of the original skin lesions after a single dose of intramuscular 2.400.000 IU penicillin G benzathine; (B) Skin lined by an orthokeratotic epidermis with spongiosis. A lymphohistiocytic inflammatory infiltrate with some scattered plasmocytes around the superficial and middle capillaries is recognized in the dermis. The arrow highlights the inflammatory infiltrate in the dermis; (C) *Treponema pallidum* antibody immunohistochemical (IHC) stain highlights the presence of spirochetes among the inflammatory cells. The arrow highlights the spirochetes among the inflammatory cells

Venereal Disease Research Laboratory (VDRL) was slightly positive with “one dilution,” and Chemiluminescence Immunoassay (CLIA) for syphilis was highly positive. Prozone phenomenon was suspected and a new diluted VDRL reported reactivity of >1:128 dilutions. Intramuscular 2.400.000 IU penicillin G benzathine was administered in a single weekly dose with a total of three doses, observing great amelioration of skin lesions (Figure 2A). A skin pathology reported spirochetes between the inflammatory cells observed in the dermis, using the *Treponema pallidum* antibody immunohistochemical (IHC) stain (Figures 2B and 2C). All of these findings confirmed that the patient was coursing with secondary syphilis. Tuberculosis was ruled out via negative bacilloscopy, culture, and PCR; bronchoalveolar lavage was negative for other infectious causes including viral and fungal etiologies. Therefore, pulmonary involvement was also attributed to secondary syphilis. The patient did not course with neurosyphilis or ocular involvement.

## DISCUSSION

The diagnosis of secondary syphilis in patients without classic mucocutaneous lesions can be challenging. Although rash involving palms, soles, or mucous membranes is characteristic, up to 15%–30% of cases may present atypical cutaneous findings, delaying recognition, and testing^1^. A case series by Muna and Shanmugam^7^ found that 47.85% of patients had palmoplantar involvement. Macules and plaques seen on palms and soles have been described as the most frequent cutaneous manifestation of secondary syphilis in the last decade^8^. In our patient, the absence of palmar, plantar, or mucosal lesions and the predominance of nonspecific maculopapular and nodular eruptions underscored the need for high clinical suspicion and comprehensive serologic evaluation.

A notable complicating factor was the prozone phenomenon, whereby exceedingly high antibody titers interfere with antigen-antibody lattice formation, yielding false-negative non-treponemal tests. This effect is reported in 0.5%–2% of secondary syphilis cases and is more frequent in settings of very high antibody levels or HIV co-infection^2,3^. Recognition of a nonreactive VDRL in the face of compatible clinical features prompted serial dilutions, ultimately revealing an VDRL titer >1:128. Failure to identify the prozone effect may postpone diagnosis and treatment^3^.

Pulmonary involvement in secondary syphilis is uncommon, with only 25 cases documented in the literature^9^. The predominant radiologic features in high-resolution chest CT are bilateral subpleural nodules, which sometimes are cavitary and, less frequently, pulmonary abscesses and halo sign^9-11^. In our patient, exclusion of tuberculosis and other infectious pathogens supported the attribution of the posterior basal tree-in-bud pattern to *Treponema pallidum* dissemination. Comparable imaging presentations have been reported, and when both non-treponemal and treponemal serologies are conclusive, lung biopsy is seldom required^12^.

The use of immunohistochemistry (IHC) for *T. pallidum* in tissue biopsies provides direct visualization of spirochetes, complementing serologic and molecular assays. Reported sensitivities of IHC in skin specimens ranged from 60% to over 80%, with specificity approaching 100%^5,6^. In our case, IHC staining of skin biopsy revealed numerous organisms between dermal inflammatory cells. Fluorescent anti-*T. pallidum* antibodies and avidin-biotin peroxidase methods have further enhanced detection, particularly in samples with low spirochetal load^6^.

## CONCLUSION

This case highlights several important considerations for clinicians and microbiologists: (1) maintain suspicion for secondary syphilis in atypical skin presentations; (2) vigilantly assess for the prozone phenomenon in non-treponemal testing; (3) recognize pulmonary involvement via characteristic CT findings and exclusion of other etiologies; and (4) use IHC as a valuable adjunctive diagnostic tool when direct detection is needed. Further studies are needed to define the prevalence of pulmonary syphilis and to optimize diagnostic algorithms incorporating serology, imaging, and tissue-based assays.

## Data Availability

The anonymized dataset generated during this study is available from the corresponding author upon reasonable request.
